# Threat of a Cycle Proof: Vertebral Artery Dissection Associated with Posterior Inferior Cerebellar Artery Infarction

**DOI:** 10.1155/2018/5387607

**Published:** 2018-12-27

**Authors:** Francisco Monteiro, Pedro Oliveira, Artur Condé

**Affiliations:** Department of Otorhinolaryngology, Head and Neck Surgery, Hospital Center of Vila Nova Gaia/Espinho, Rua Conceição Fernandes s/n, 4434-502 Vila Nova de Gaia, Portugal

## Abstract

This paper presents a case of a perfectly healthy 36-year-old male, who went to the emergency department with a clinical picture of diffuse headache, dizziness, and asthenia with 3 days of evolution, after a long cycling race. He was admitted to the ENT Department with suspected diagnosis of peripheral vertigo. The patient developed hypoesthesia of the face, diplopia, right lateropulsion, and Romberg with preferential rightward fall, and imaging studies demonstrated an extracranial vertebral artery dissection concomitant with PICA territory infarction. This is a rare described case of a vertebral artery dissection concomitant with an infarction of the PICA territory. This case demonstrates the importance of maintaining a high degree of suspicion of stroke in patients with signs/symptoms of nystagmus/vertigo and the relevance of magnetic resonance imaging instead of tomography in the detection of these serious clinic conditions.

## 1. Introduction

Vertebral artery dissection (VAD) is a relatively rare event but a major cause of stroke in young and middle-aged patients [1]. Only twenty percent of ischemic events occur in the posterior circulation due to atherosclerosis, arterial embolization, or arterial dissection [2]. The concomitance of vertebral arterial dissection associated with infarction of the territory of the posterior inferior cerebellar artery (PICA) is an even rarer event. The truth is that this young man who had been on a three-hour bike ride went to the emergency department with a common complaint of vertigo and presented a rare association of a vertebral artery dissection with PICA infarction.

## 2. Case Presentation

The authors report a case of a 36-year-old active man with no relevant medical history, who went to the emergency department due to a diffuse headache and dizziness, with a 3-day course after a long bicycle ride. The patient referred that these symptoms were usual after an intense physical activity as he regularly performed in cycle races. He was admitted to Neurology observation in the Emergency Room (ER). On examination, there were no evident *de novo* neurological signs. A cerebral CT was performed, and it was normal. He was then referred to Ear, Nose, and Throat (ENT) observation due to suspicion of peripheral vertigo.

**Figure 1:**
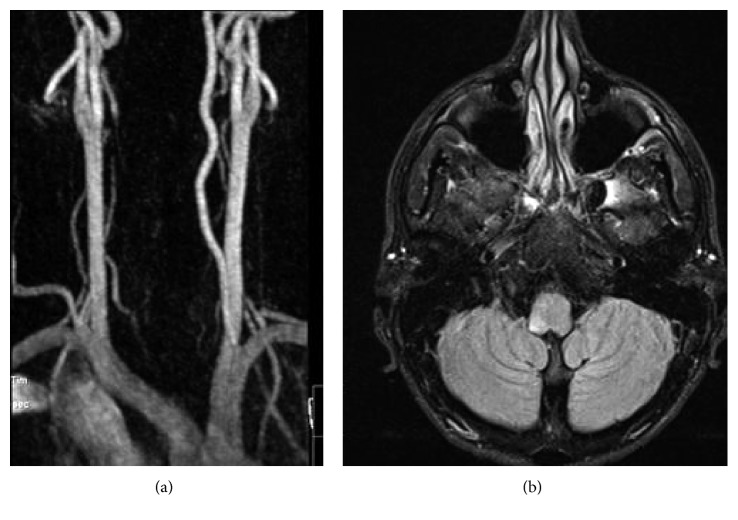
(a) Asymmetry at V2 segment of the left vertebral artery compared to that of the right vertebral artery (red arrow); (b) hyperintense signal in the right dorsolateral segment of the bulb, corresponding to the PICA infarction territory.

The ENT examination revealed a horizontal-rotatory nystagmus, with rapid phase to the right, that was exhaustible in the gaze, however with a normal head impulse test. The Neurology ER team assumed noncentral vertigo since at this time there was no evidence of any signs of a central cause, neither in physical examination nor in the imaging test performed. This diagnosis seemed the most likely to the team. He was admitted to the ENT ward, and symptomatic and medical treatment was initiated. There was clinical stabilization until the 3^rd^ day, when sudden symptoms and signs emerged: ipsilateral downward fall, right hemifacial paresthesia, right hemifacial pain, ipsilateral limb ataxia with ataxic gait, and diplopia. An emergent magnetic resonance angiography revealed “(…) hyperintense area in T2 and T2 FLAIR in the dorsal lateral aspect of the right bulb that in the diffusion study showed a marked restriction (…).” In the arteriography study, it was identified that “an occlusion of the right vertebral artery was identified in segment V2, after showing progressive reduction and contour irregularity” (Figure 1).

The patient was transferred to the Cerebrovascular Accidents Unit (CVAU) and started treatment with antiplatelet therapy, rehabilitation with obvious improvement. At the time of hospital discharge (15 days after admission), he had just a slight alteration in gait and left eye ophthalmoparesis.

## 3. Discussion

Vertebral artery dissection is a relatively rare event, with an annual incidence of approximately 1–1.5 cases/100,000 [3]. Headache and neck pain are important warning signs that most often precede neurological symptoms [4]. Neurological sequelae of extra- or intracranial vertebral artery disease may result from cerebral ischemia due to thromboembolism, hypoperfusion, or a combination of both. However, thromboembolism is now considered the major cause of ischemic events [5]. In our patient, the vertebral dissection was associated with extenuating physical activity (after an intense cycling ride), and signs and symptoms fitted Wallenberg's syndrome. Pathophysiology of this delayed neurological scenario may be explained due to reduced blood flow at the PICA by either a dislodged embolus from the vertebral dissected segment with consequent arterial occlusion or a flow decrease as a consequence of arterial dissection. Given the good clinical evolution, with resolution of almost all the clinical signs that were compatible with Wallenberg's syndrome, we can assume that collateral circulation of the anterior inferior cerebellar artery would probably have enhanced the clinical recovery of the patient, with consequent reperfusion of the dorsal lateral region of the bulb. To our knowledge, there is not yet a case described on PubMed of an association of a vertebral arterial dissection with an infarction of the posterior inferior cerebellar artery territory. So, the apparent simple initial case of dizziness was hiding a more complex diagnosis like a vertebral dissection, as its progression caused a posterior inferior cerebellar artery infarction.

## 4. Conclusion

This case demonstrates the importance of raising awareness of possibility of stroke even in young patients developing nystagmus/vertigo, even with normal imaging test and no relevant signs at the initial neurological examination. On the one hand, this case reinforces the relevance of magnetic resonance imaging as the most sensitive test in the detection of these serious clinic conditions. On the other hand, it raises the importance of close follow-up of these patients since an initial scarce clinical scenario may reveal a life-threatening picture, such as a cerebral infarction as a consequence of a vertebral dissection.
